# 1130. Ensovibep antiviral activity in ambulatory patients with COVID-19 is independent of baseline anti-SARS-CoV-2 antibodies and exhibits minimal selective pressure – Results from the placebo-controlled EMPATHY trial

**DOI:** 10.1093/ofid/ofac492.969

**Published:** 2022-12-15

**Authors:** Luis Abrishamian, Marc Bonten, Richa Chandra, Damodaran Solai Elango, Pierre Fustier, Kinfemichael Gedif, Susana Goncalves, Awawu Igbinadolor, Jeff Kingsley, Charles G Knutson, Petra Kukkaro, Nagalingeswaran Kumarasamy, Philippe Legenne, Martha Mekebeb-Reuter, Krishnan Ramanathan, Evgeniya Reshetnyak, Michael Robinson, Jennifer Rosa, Marianne Soergel, Vaia Stavropoulou, Nina Stojcheva, Michael T Stumpp, Andreas Tietz, Xiaojun Zhao, Zhaojie Zhang

**Affiliations:** South Bay Clinical Research Institute, Redondo Beach, CA, USA, Redonda Beach, California; Julius Center for Health Sciences and Primary Care, University Medical Center, Utrecht, Utrecht, Netherlands; Novartis Pharmaceuticals Corporation, East Hanover, NJ, USA, East Hanover, New Jersey; Novartis Healthcare Pvt Ltd, Hyderabad, India, Hyderabad, Telangana, India; Molecular Partners AG, Zurich-Schlieren, Switzerland, Zurich-Schlieren, Zurich, Switzerland; Novartis Pharmaceuticals Corporation, Fort Worth, TX, USA, Fort Worth, Texas; Novartis Pharma AG, Basel, Switzerland, Basel, Basel-Stadt, Switzerland; Monroe Biomedical Research, Monroe, NC, USA, Monroe, North Carolina; Centricity Research, Columbus, GA, USA, Columbus, Georgia; Novartis Institutes for BioMedical Research, Cambridge, MA, USA, Cambridge, Massachusetts; Novartis Pharma AG, Basel, Switzerland, Basel, Basel-Stadt, Switzerland; VHS Infectious Diseases Medical Centre, Chennai Antiviral Research and Treatment Clinical Research Site, Chennai, India, Chennai, Tamil Nadu, India; Molecular Partners AG, Zurich-Schlieren, Switzerland, Zurich-Schlieren, Zurich, Switzerland; Excellentis Clinical Trial Consultants, George, South Africa, George, Western Cape, South Africa; Novartis Pharma AG, Basel, Switzerland, Basel, Basel-Stadt, Switzerland; Novartis Pharmaceuticals Corporation, East Hanover, NJ, USA, East Hanover, New Jersey; Novartis Institute for Tropical Disease (NITD), Emeryville, CA, USA, Emeryville, California; Clinresco Centres, Gauteng, South Africa, Gauteng, Gauteng, South Africa; Molecular Partners AG, Zurich-Schlieren, Switzerland, Zurich-Schlieren, Zurich, Switzerland; Molecular Partners AG, Zurich-Schlieren, Switzerland, Zurich-Schlieren, Zurich, Switzerland; Molecular Partners AG, Zurich-Schlieren, Switzerland, Zurich-Schlieren, Zurich, Switzerland; Molecular Partners AG, Zurich-Schlieren, Switzerland, Zurich-Schlieren, Zurich, Switzerland; Novartis Pharma AG, Basel, Switzerland, Basel, Basel-Stadt, Switzerland; Novartis Institutes for BioMedical Research, Cambridge, MA, USA, Cambridge, Massachusetts; Novartis Institutes for BioMedical Research, Cambridge, MA, USA, Cambridge, Massachusetts

## Abstract

**Background:**

Ensovibep is a multi-specific DARPin (designed ankyrin repeat protein) antiviral in clinical development for treatment of COVID-19. In the Phase 2 EMPATHY study, ensovibep demonstrated greater viral load decline versus placebo. Here we report (1) the efficacy of ensovibep in patients with and without anti-SARS-CoV-2 antibodies at baseline and (2) SARS-CoV-2 mutation emergence data with treatment.

**Methods:**

Eligible ambulatory patients with ≥2 COVID-19 symptoms (onset within 7 days) and positive SARS-CoV-2 rapid antigen test on day of dosing, were randomized (1:1:1:1) to ensovibep (600, 225 or 75 mg) or placebo as single, IV infusion. Chemiluminescent immunoassays were used for antibody detection (SARS-CoV-2 S1/S2 IgG and SARS-CoV-2 IgM). A pre-specified subgroup analysis was performed based on baseline anti-SARS-CoV-2 antibody status. Analysis of changes in viral genome from baseline to post baseline was performed to evaluate treatment-emergent mutations.

**Results:**

Of the patients analyzed, 48.5% had anti-SARS-CoV-2 antibodies at baseline. Baseline log_10_ SARS-CoV-2 viral load (mean ±SD) was similar across groups [ensovibep (all doses) 6.5 ±1.5, placebo 6.2 ±1.5]; > 90% were infected with the Delta (B.1.617.2) variant. SARS-CoV-2 viral load reduction up to Day 8 showed similar effects in favor of ensovibep compared with placebo regardless of the presence of anti-SARS-CoV-2 antibodies (**Figure 1**). Patients in ensovibep 75 mg, 600 mg, and placebo groups had comparable incidences of emergent mutations, with a higher incidence in the 225 mg group. Based on analysis of 70% of the expected viral sequencing data, two mutations in the key binding residues of ensovibep were observed (Y489H and F486L) in a total of three patients treated with ensovibep. These patients either cleared virus by Day 8 or mutations were transient (occurred at a single time point but not later in the course of infection).

**Figure 1:**
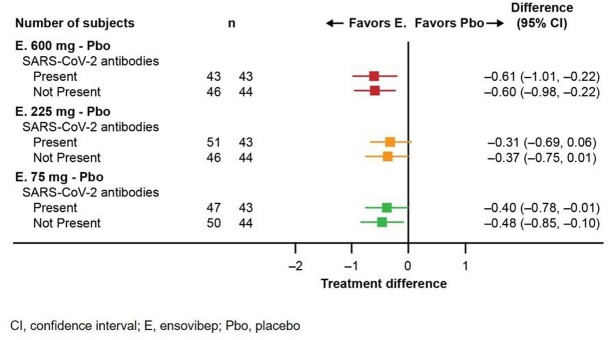
Forest plot of estimated treatment differences and associated 95% confidence intervals in time-weighted change from baseline in log10 SARS-CoV-2 viral load through Day 8 by subgroups for the presence of anti-SARS-CoV-2 antibodies (SARS-CoV-2 S1/S2 IgG and/or SARS-CoV-2 IgM) at baseline.

**Conclusion:**

Ensovibep effectively reduces SARS-CoV-2 viral load regardless of the presence of anti-SARS-CoV-2 antibodies at baseline. Furthermore, there were no emerging mutations of concern, indicating that a single dose administration of ensovibep is associated with minimal selective pressure.

**Disclosures:**

**Marc Bonten, MD, PhD**, Astra-Zeneca: Advisor/Consultant|Janssen: Advisor/Consultant|Merck: Advisor/Consultant|Novartis: Advisor/Consultant **Richa Chandra, MD**, Novartis Pharmaceuticals Corporation: Employee **Damodaran Solai Elango, MD**, Novartis Healthcare Pvt Ltd: Employee **Pierre Fustier, PhD**, Molecular Partners AG: Employee **Kinfemichael Gedif, PhD**, Novartis Pharmaceuticals Corporation: Employee **Susana Goncalves, MD**, Novartis Pharma AG: Employee **Awawu Igbinadolor, MD**, Novartis: Awawu Igbinadolor reports financial support from different pharmaceutical companies and organizations **Jeff Kingsley, DO, MBA, CPI, FACRP**, Centricity Research: Other **Charles G. Knutson, PhD**, Novartis Institutes for BioMedical Research: Employee **Petra Kukkaro, PhD**, Novartis Pharma AG: Employee **Nagalingeswaran Kumarasamy, MD**, Novartis: Nagalingeswaran Kumarasamy reports financial support from different pharmaceutical companies and organizations **Philippe Legenne, MD**, Molecular Partners AG: Employee **Martha Mekebeb-Reuter, MD**, Novartis: Martha Mekebeb-Reuter reports financial support from different pharmaceutical companies and organizations **Krishnan Ramanathan, MD**, Novartis Pharma AG: Employee **Evgeniya Reshetnyak, PhD**, Novartis Pharmaceuticals Corporation: Employee **Michael Robinson, PhD**, Novartis Institute for Tropical Disease: Employee **Jennifer Rosa, MD**, Novartis: Jennifer Rosa reports financial support from different pharmaceutical companies and organizations **Marianne Soergel, MD**, Molecular Partners AG: Employee **Vaia Stavropoulou, PhD**, Molecular Partners AG: Employee **Nina Stojcheva, PhD**, Molecular Partners AG: Employee **Michael T. Stumpp, PhD**, Molecular Partners AG: Employee **Andreas Tietz, MD**, Novartis Pharma AG: Employee **Xiaojun Zhao, PhD**, Novartis Institutes for BioMedical Research: Employee **Zhaojie Zhang, PhD**, 8. Novartis Institutes for BioMedical Research: Employee.

